# Current State of the Art in Neurosurgical Operative Microscopes

**DOI:** 10.7759/cureus.104305

**Published:** 2026-02-26

**Authors:** Kivanc Yangi, Egemen Gok, Michell Goyal, Pravarakhya Puppalla, Mark Preul

**Affiliations:** 1 Neurosurgery, Barrow Neurological Institute, Phoenix, USA; 2 Medicine, Creighton University, Phoenix, USA; 3 Ophthalmology, Retina Associates Tucson, Tucson, USA

**Keywords:** microneurosurgery, microscope, microscopic neurosurgery, microscopic surgery, neurosurgery, scope, surgical microscope

## Abstract

Surgical microscopes play a critical role in neurosurgery, providing high-resolution visualization, magnification, and ergonomic advantages that support precise surgical interventions and enhance the training environment. Recent technological advances have further expanded their clinical value. This study evaluated the current generation of neurosurgical operative microscopes, identifying and comparing the technical specifications, advantages, and limitations of the latest models released by six leading manufacturers: Carl Zeiss Meditec AG (Jena, Germany); Leica Microsystems GmbH (Wetzlar, Germany (a subsidiary of Danaher Corp., Washington, DC); Mitaka Kohki Co., Ltd. (Tokyo, Japan); Olympus Corp. (Tokyo, Japan); Aesculap, Inc. (Tuttlingen, Germany (a subsidiary of B. Braun Group, Melsungen, Germany)); and Synaptive Medical, Inc. (Ontario, Canada). Information was gathered through manufacturer-provided technical data, direct communication with company representatives, and published reports on neurosurgical microscopes. Particular attention was given to models in use worldwide. A total of 21 surgical microscopes and exoscopes from six leading manufacturers were reviewed. Zeiss microscopes, particularly the Kinevo 900 and Pentero series, demonstrated advanced integration with robotic positioning, compatibility with image-guided surgery, and automated movement systems. Leica models were notable for their emphasis on surgical education and training, with systems such as the M320 T microscope, the MyVeo headset, and augmented reality (AR)-enhanced fluorescence modules. Mitaka microscopes were distinguished by their lightweight design and compact stands, offering enhanced mobility in operating rooms with limited space. The specialized exoscope models from Olympus, Aesculap, and Synaptive offered excellent image clarity and ergonomic control. Notably, refurbished older microscopes are often used in low- and middle-income countries due to financial constraints. Contemporary neurosurgical microscopes have evolved into multifunctional platforms that support surgical precision and enhance training, education, and intraoperative visualization. The integration of cutting-edge technologies (e.g., AR visualization, artificial intelligence, robotics, wireless controls, and adaptive viewing platforms) signals a promising path for the future of surgical innovation. However, global variability in model availability reflects underlying resource disparities. Continued innovation and efforts to make advanced technologies more accessible are essential to supporting neurosurgeons worldwide.

## Introduction and background

Operating microscopes are an indispensable component of modern neurosurgical practice. Since their introduction, improvements in illumination and magnification have enabled safe access to deep and previously inaccessible surgical targets, marking a key technological milestone in the evolution of microneurosurgery. Over the decades, different manufacturers have developed a wide range of microscope models, each tailored to distinct surgical needs and workflow environments. As a result, contemporary neurosurgical practice worldwide incorporates a spectrum of operative microscopes, from older, long-standing models still in use in many low- and middle-income countries (LMICs) to the most advanced artificial intelligence (AI)-integrated platforms emerging in high-resource centers.

This state-of-the-art review aims to consolidate, in a single resource, the currently available neurosurgical operating microscopes, from widely used earlier-generation systems to the latest models integrating AI, augmented visualization, and advanced digital workflows. There is a clear gap in the literature: no comprehensive, up-to-date source details the technical specifications of these microscopes or compares their optical designs, illumination systems, ergonomic features, accessory compatibility, and maintenance considerations. Because much of this information is not comprehensively reported in the published literature, it cannot be reliably obtained through database-driven systematic searches. Accordingly, primary technical data for this review were sourced directly from manufacturers. This review should not be construed as a marketing tool or with any sort of financial incentive. In most cases, manufacturers were responsive to our requests for information. The objective of this work is not to present a traditional systematic review, but rather to provide a rigorously compiled, manufacturer-verified, and technically accurate reference on operating microscopes currently used in neurosurgery.

Early microscope development (1920s-1970s)

Although the surgical microscope was first introduced in 1921, its use in neurosurgery began in 1957 when Theodore Kurze (1922-2002) pioneered the first neurosurgical operating microscope in California [1]. Since then, major technological improvements have enhanced magnification, illumination, and ergonomics, contributing to greater surgical precision and improved patient outcomes [2,3]. Early refinements included customized and later motorized stands for Carl Zeiss microscopes (now Zeiss Meditec AG, Jena, Germany) beginning in 1958, followed by the integration of additional visualization accessories, such as short ocular tubes and improved optical components, culminating in early models like the Zeiss OPMI 1 [4,5]. In 1964, Zeiss introduced the diploscope, which allowed simultaneous visualization of the operative field and facilitated collaborative surgery, greatly benefiting resident training [4]. Its ergonomic overhang design also reduced surgeon fatigue during prolonged procedures, contributing to decreased musculoskeletal strain [6].

Robotics and image-guided surgery (1980s-present)

The integration of robotics and other advanced features into surgical microscopy during the 1980s and 1990s further revolutionized surgical practice. Robotic systems offered key advantages, such as tremor filtering, motion scaling, and force sensing, that enabled greater efficiency, enhanced precision, and minimal invasiveness [7]. Robots were first used in neurosurgical procedures in 1985. The earliest known robotic-assisted neurosurgical intervention was performed by Yik San Kwoh using the computed tomography (CT)-guided PUMA (Programmable Universal Machine for Assembly) 200 robot (developed by Westinghouse Electric Co., Pittsburgh, PA) [7-9].

Subsequent developments followed rapidly. In 1989, the Neuromate surgical robot (developed by Renishaw plc, New Mills, UK) was used in CT-guided frameless stereotactic procedures. In 1991, the Minerva robot introduced by the University Hospital Center, Lausanne, Switzerland, achieved real-time correction of brain shift under intraoperative CT guidance [7,10,11]. In 1995, the Jet Propulsion Laboratory at the California Institute of Technology (Pasadena, CA), in collaboration with MicroDexterity Systems, Inc. (Albuquerque, NM), developed the Robot-assisted Microsurgery System (RAMS) using technology from the US National Aeronautics and Space Administration. RAMS replaced CT guidance with magnetic resonance imaging (MRI)-based localization, marking a shift toward MRI-compatible robotics. This transition was further accelerated by the introduction of intraoperative MRI at Brigham and Women’s Hospital in the mid-1990s, which sparked widespread interest in image-guided neurosurgery (IGS) [12]. Research into MRI-compatible neurosurgical robots gained momentum, and in 1995, the School of Medicine at the University of Tokyo (Matsumoto, Japan) developed the first magnetic resonance-compatible neurosurgical robot (NeuRobot) for stereotactic biopsy [7].

Another key development was the Zeiss-MKM microscope, a computer-assisted surgical microscope equipped with a frameless navigation system that was designed for IGS procedures [13,14]. This system provided high spatial accuracy, enabling accurate data alignment and serving as a valuable tool for validating functional MRI findings [14]. The primary advantage of this system was its ability to enable preoperative lesion contouring on a computer workstation that could be projected onto the patient’s head in the operating room before surgery.

One of the first systems to integrate real-time image guidance with surgical microscopy was the Elekta SurgiScope (Elekta Instruments, Inc., Stockholm, Sweden), which was developed in the 1990s [15]. The SurgiScope greatly enhanced precision in neurosurgical procedures by incorporating robotic control into the operating microscope, marking an advancement in integrating frameless stereotaxy with microsurgical techniques. This system enabled preoperative planning based on imaging data, allowing the microscope to follow predefined trajectories during surgery. It also featured laser localization to precisely identify the target entry point, and it could be continuously repositioned to aid lesion resection. However, its technical complexity, which required specialized training of the surgical team, was a drawback [15].

Fluorescence and digital integration (2000s-present)

In contrast, the concept of fluorescence-guided surgery (FGS) has gained increasing prominence in neurosurgery, as it facilitates the differentiation of tumor tissue from surrounding noninfiltrated healthy brain parenchyma and enables visualization of the cerebral vasculature. This technique originated in 1948, when Moore et al. [16] proposed that intracranial neoplasms could be better localized using fluorescein. In 1982, Murray [17] used fluorescence to help determine the extent of tumor resection. However, widespread adoption of FGS began only after Stummer et al.’s [18] landmark randomized controlled trial of 5-aminolevulinic acid (5-ALA) in high-grade gliomas, the results of which were published in 2006. Over the past two decades, continued advances in FGS have included the development of various fluorophores and the integration of optimized filters by leading surgical microscope manufacturers [19].

Artificial intelligence and hybrid systems (2020s-present)

The integration of AI has further accelerated advances in operative microscope technology in the 2020s [20]. A notable example of this advancement is the Kinevo 900 S (launched by Zeiss in 2024) [21-31]. This model features an integrated AutoCenter function powered by an AI-trained algorithm. The AutoCenter function automatically centers the area of interest within the surgeon’s field of view during the procedure. It recognizes surgical tools and automatically centers the field of view on the tips of the instruments. The AutoCenter function enables hands-free positioning, which eliminates the need for repeated manual microscope adjustments that are often required in small, deep surgical fields.

Functions like enhanced magnification, three-dimensional (3D) imaging, fluorescence modes, illumination, and robotic assistance have enabled meticulous neurosurgical procedures. As a result, specialized and more optimized neurosurgery has become possible to address brain tumors, cerebrovascular pathologies, deep-seated lesions, and spine conditions, thereby improving patient outcomes. Neurosurgical microscope technology continues to evolve through close collaboration among neurosurgeons and manufacturers. The operating microscope for neurosurgery has evolved into a multifunctional imaging platform.

In this milieu of rapid technological development, we conducted this review to provide information on the most up-to-date neurosurgical microscope models from six leading manufacturers, along with summaries of their technical specifications, advantages, and disadvantages. Our purpose is not to endorse a particular neurosurgical operating microscope model or manufacturer but rather to coalesce publicly available information from academic sources and company documents and websites, which may not be exhaustive. Because it is not feasible to list every model and version of the surgical microscopes in use worldwide, we focused on the most recently launched models and versions offered by leading manufacturers. Additionally, because it is not possible to review every individual feature of these microscopes in a study, we have highlighted only their most prominent features. No financial or marketing arrangements with any manufacturer were made for this article.

## Review

Current state-of-the-art neurosurgical microscopes by leading microscope brands

In addition to Zeiss, there are five other major surgical microscope brands used in daily neurosurgical practice: Leica Microsystems GmbH (Wetzlar, Germany (a subsidiary of Danaher Corp., Washington, DC)); Mitaka Kohki Co., Ltd. (Tokyo, Japan); Olympus Corp. (Tokyo, Japan); Aesculap, Inc. (Tuttlingen, Germany (a subsidiary of B. Braun Group, Melsungen, Germany)); and Synaptive Medical, Inc. (Mississauga, Ontario, Canada). Although these manufacturers have introduced numerous microscope models over the years, most are now considered outdated; many older models nevertheless remain in active use worldwide, particularly in LMICs. Collaborations between some LMICs and high-income countries have resulted in the use of refurbished versions of older models in specific LMIC settings [32]. This review of a wide range of neurosurgical microscope models in clinical use does not cover every model worldwide. Instead, it focuses on the most recent and most widely used models in neurosurgery.

Zeiss (Carl Zeiss Meditec AG)

Zeiss, founded by Carl Zeiss in 1846, is a well-known company shaped by its history in optical and industrial precision standardization. The Zeiss OPMI 1, developed in 1953, was the first surgical microscope to be used in neurosurgery, employed by Theodor Kurze in 1957 at the University of Southern California [4]. With the aid of the microscope, Kurze removed a neurilemoma from the facial nerve (cranial nerve VII) in a five-year-old patient. Subsequent modifications to the Zeiss OPMI 1 from 1959 to 1964 included the addition of an electric-hydraulic chair, a motorized zoom objective, surgeon armrests, a patient headrest, ocular tubes, and a slit lamp. In 1965, the OPMI 2 was released; it featured motorized zoom and focus. The following year, the OPMI 3 was released; it featured a rotating prism, suturing reticules in the eyepieces, and a sterilization device. Subsequent models, such as the OPMI 4, OPMI 5, and OPMI 7P/H, provided deeper field focusing, were more compact, and enabled multiple surgeons to work simultaneously. In 1991 and 1994, respectively, Zeiss released the OPMI CS and the OPMI ES, which were explicitly designed for neurosurgery. In 1997, the OPMI Neuro was released; it came equipped with Multivision, which provides advanced imaging directly into the eyepieces [4]. In the 2010s, Zeiss began developing microscope models integrated with a point-lock system that maintains a fixed focal point on a designated target by adjusting the angle and focal length as the microscope is moved, thereby enabling the definition of a surgical trajectory from a selected entry point [33,34].

Current Zeiss models: Zeiss has introduced many microscope models with different features over the years. The most recent Zeiss neurosurgical microscopes, as of March 15, 2025, are the Kinevo 900 S [27] and the Pentero 800 S (Table 1) [21-31].

**Table 1:** Technical specifications of surgical microscopes from Zeiss, Olympus, and Aesculap auto: automatic, BL: blue light, Exo: exoscope, LED: light-emitting diode, Micro: microscope, NA: not applicable, NIR: near-infrared, NS: not specified, V: volts, VA: volt-ampere. ^a^Hybrid: a microscope equipped with digital three-dimensional functionality, allowing it to operate both as a traditional optical microscope and as an exoscope.

Feature	Zeiss								Olympus	Aesculap
	OPMI Neuro NC-4 [28]	OPMI Pentero [25]	Pentero 900 [26]	Pentero 800 [23]	Kinevo 900 [27]	Tivato 700 [24]	Pentero 800 S [22]	Kinevo 900 S [21]	ORBEYE [29,30]	Aeos [31]
Type	Micro	Micro	Micro	Micro	Hybrid^a^	Micro	Hybrid^a^	Hybrid^a^	Exo	Exo
Power consumption	100–240 V	100–240 V	100–240 V	100–240 V	100–240 V	100–240 V	100–240 V	100–240 V	NS	NS
	50–60 Hz	50–60 Hz	50–60 Hz	50–60 Hz	50–60 Hz	50–60 Hz	50–60 Hz	50–60 Hz		
	1.380 VA	1.200 VA	1.200 VA	1.200 VA	1.350 VA	800 VA	1.200 VA	1.500 VA		
Weight, kg	310	325	358	365	395	395	365	395	216	206
Magnification	2.2× to 13.4× with 10× eyepiece	1.1× to 13.0× with 10× eyepiece	4.0× to 24.0× with 10× eyepiece	4.0× to 24.0× with 10× eyepiece	NS	4.0× to 24.0× with 10× eyepiece	2.2× to 13.0× with 10× eyepiece	4.0× to 24.0× with 10× eyepiece	13× optical, 2× monitor (total 26×)	10× optical Z
Eyepieces	10×	10×	10×	10×	10×	10×	10×	10×	NA (exo only, no oculars)	NA (exo only, no oculars)
		12.5×	12.5×	12.5×	12.5×	12.5×	12.5×	12.5×		
Fluorescence dyes	None	None	Infrared 800	Infrared 800	Blue 400	Yellow 560	Infrared 800	Blue 400	BL imaging mode, NIR (ICG)	DUV400, DIR800
			Flow 800	-	Yellow 560	Yellow 560	Flow 800	Blue 400 S		
			Blue 400	-	Infrared 800	LED Infrared 801	Yellow 560	Yellow 560		
			Yellow 560	-	Flow 800	-	Blue 400	Infrared 800		
			-	-	-	-	-	Flow 800		
Illumination	300-W xenon lamp, 100-W halogen backup, adjustable brightness (light guide (Superlux 301))	Dual 300-W xenon lamps (light guide (Superlux 330))	Dual 300-W xenon lamps (light guide (Superlux 330))	Dual 300-W xenon lamps (light guide (Superlux 330))	2×300-W xenon, motorized shadow light	TriLED integrated xenon lights, with auto lamp exchange	Fully integrated xenon light with auto lamp exchange	Fully integrated 300-W xenon light	LED	2× coaxial LED
Working distance, mm	195–420	200–500	200–500	200–500	200–625	200–500 or 200–625	200–500 or 200–625	200–625	220–550	200–450

**Figure 1:**
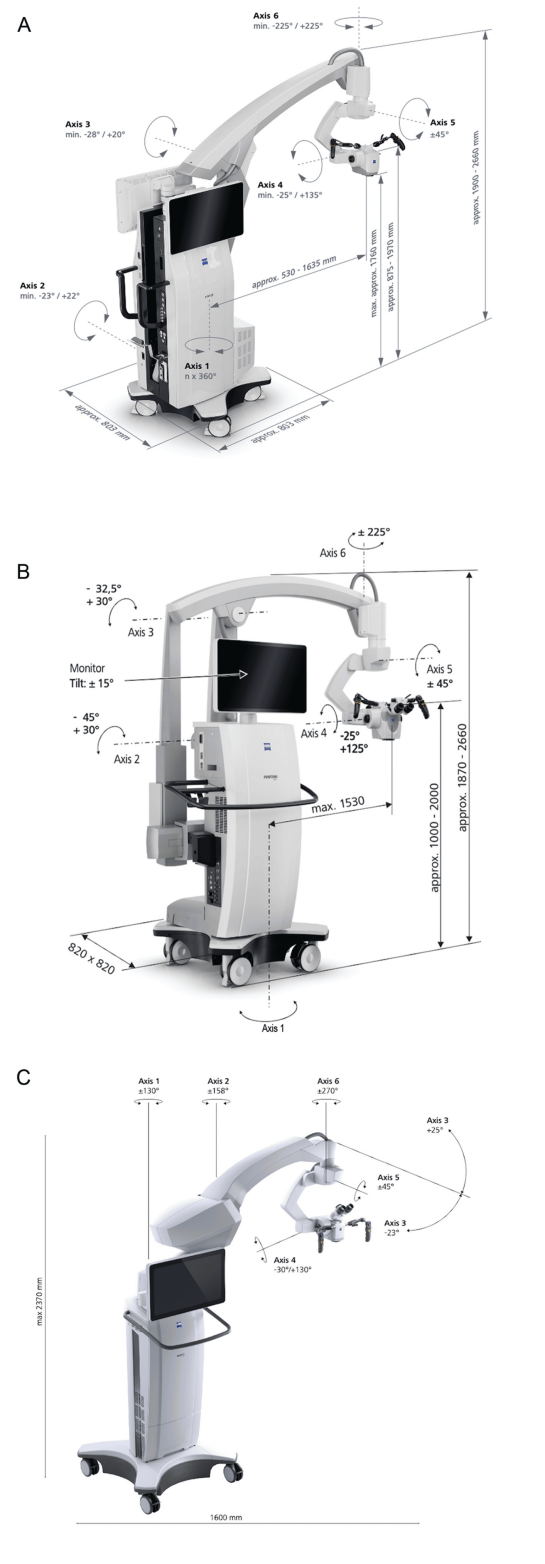
Currently available microscope models by Carl Zeiss Meditec AG (Jena, Germany) that are actively used in neurosurgery (A) Kinevo 900 S [21]. (B) Pentero 800S [22]. (C) Tivato 700 [24]. Used with permission from Carl Zeiss Meditec.

The Kinevo 900 S is an upgraded version of an earlier model (Kinevo 900) that features advanced digital visualization, robotic assistance, and connected intelligence (Figure 1A) [21]. It includes Zeiss Blue 400 S, an angiographic visualization mode that employs fluorescence technology to visualize high-grade gliomas with minimal changes across illumination modes. For targeted vascular visualization, it supports fluorescence options such as Infrared 800, Flow 800, Yellow 560, and Blue 400 S. The Flow 800 module highlights fluorescence-stained tissues, whereas nonstained areas remain natural in appearance.

The Kinevo 900 S incorporates AI through features such as AutoCenter, Voice Assistant, and collaborative robotics (Cobotic Assistant, PointLock, and PositionMemory), thereby enabling automatic centering, instrument-tip localization, voice-command integration, and enhanced surgical workflow efficiency. With the PositionMemory feature, previously stored configurations for the robot's position, path, focus, and zoom can be retrieved instantly at any stage of the procedure [35]. Workflow improvements are enabled by the Cobotic Assistant, which automatically centers the focus, switches fluorescence modes, and captures images and videos. Additional features include a motorized z-axis and integrated data management tools. Other benefits of the Kinevo 900 S include 45° endoscopic visualization with the QEVO microinspection tool, precise robotic control, digital overlays in the surgical field, and seamless integration with other systems [36]. However, the cost and complexity of the Kinevo 900 S require a substantial financial investment and technical training for its practical use.

The Zeiss Pentero 800 S is widely used across surgical specialties, including neurosurgery (Figure 1B, Table 1) [22]. It features a fully integrated 4K 3D camera, QEVO inspection tool for angled views, and digital hybrid visualization for switching between digital and optical views. A resolution enhancer provides 60% magnification and a 50% increase in resolution (up to 120 lp/mm), supporting delicate procedures. The system includes fluorescence modules (Infrared 800, Flow 800 option, Yellow 560, Blue 400), and it supports data connectivity across systems, similar to the Kinevo 900 S.

Benefits of the Pentero 800 S include high-quality optics, enhanced depth-of-field control, integrated fluorescence imaging, and reliable performance across diverse surgical applications [36]. However, it lacks certain advanced digital and robotic features of the Kinevo 900 S and may require modular upgrades to match its capabilities.

There are other Zeiss microscope models. The Tivato 700 is an advanced neurosurgical visualization system featuring 4K imaging, SpeedFokus (single-button automatic focus adjustment) for sharper images, and smart connectivity (Figure 1C, Table 1) [24]. It includes ergonomic features, such as a long microscope arm, vibration damping, and the automated features AutoBalance and AutoDrape. The Tivato 700 supports an efficient intraoperative workflow through its fluorescence options (Infrared 800 and Yellow 560 light-emitting diode (LED)), TriLED and xenon lighting, navigation integration, and connectivity to PACS (picture archiving and communication system) and DICOM (digital imaging and communications in medicine).

**Figure 2:**
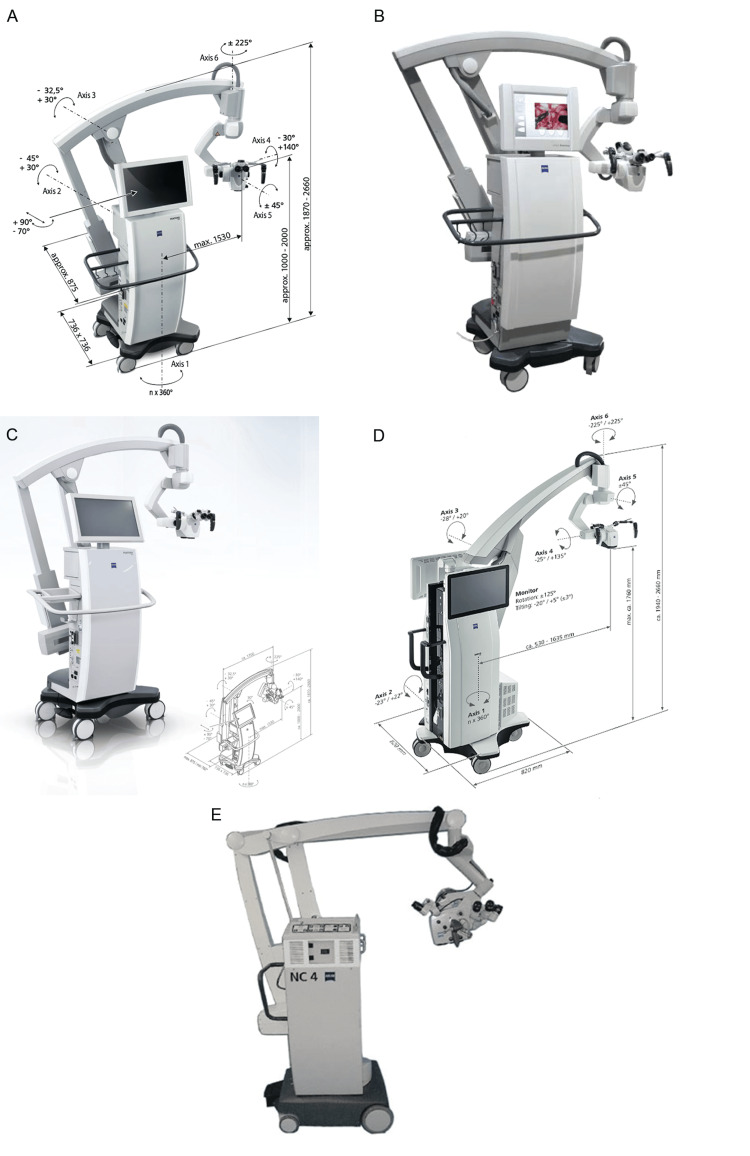
Earlier models of microscopes from Carl Zeiss Meditec AG (Jena, Germany) that continue to be used worldwide in neurosurgical practice (A) OPMI Pentero 800 [23]. (B) OPMI Pentero [25]. (C) Pentero 900 [26]. (D) Kinevo 900 [27]. (E) OPMI Neuro NC-4 [28]. Used with permission from Carl Zeiss Meditec.

The Pentero 800 provides 39× magnification, a double-iris diaphragm, and patented two-channel xenon illumination for real-time fluorescence clarity (Infrared 800) (Figure 2A, Table 1) [23]. The OPMI Pentero includes digital visualization, MultiVision, and automated features like AutoBalance and AutoDrape, with DICOM and touchscreen operation (Figure 2B) [25]. The Pentero 900 builds on this functionality by incorporating apochromatic optics, video integration, and enhanced ergonomics, including a wireless foot control and dynamic tube adjustment (Figure 2C) [26].

The Kinevo 900 integrates robotics, digital hybrid visualization, and full fluorescence support (Blue 400, Yellow 560, and Infrared 800 with Flow 800) (Figure 2D) [27]. Its 4K imaging, QEVO micro-inspection tool, voice and foot controls, and hospital connectivity enhance surgical precision and user comfort. The Zeiss OPMI Neuro NC-4, a precursor to the Pentero line, features autofocus, a heads-up display, and integration with platforms like Brainlab SE and Medtronic PLC (Figure 2E, Table 1) [28]. The OPMI Neuro NC-4 remains in use globally because it offers high-quality optics and ergonomic controls and is less costly than newer models.

Features of Zeiss neurosurgical microscopes: Zeiss microscopes, such as the Kinevo 900 S, Pentero series, and Tivato 700, support intraoperative fluorescence, including Blue 400, Blue 400 S, Yellow 560 LED, and Infrared 800 with Flow 800. Furthermore, some models, such as the Kinevo 900, feature an integrated QEVO micro-inspection tool. This tool supports deep-corridor access, aiding extensive tumor resection; however, it requires careful use due to its extended reach and angled optics [36,37]. The Kinevo 900 S is one of Zeiss’s latest models, distinguished by its AI integration, instrument tip-tracking capability, and voice command functionality. This model also supports head-mounted displays. For example, the CR3 (HMDmd) wearable surgical monitor provides high-definition two-dimensional (2D) and 3D imaging with direct plug-and-play compatibility for the Kinevo 900 S [21,38]. The CR3 features a LookDown function that enables surgeons to view their hands and instruments without excessive head movement while preserving peripheral vision. In summary, Zeiss surgical microscopes excel in neurosurgery by combining advanced optical performance and digital integration, continually setting new standards for surgical visualization and precision.

Leica (Leica Microsystems GmbH)

Established in 1849 as Carl Kellner’s Optical Institute in Wetzlar, Germany, Leica began as a family-run business dedicated to optical excellence [39]. After Ernst Leitz joined the company as an engineer in 1865, he took over in 1869, and the company rapidly expanded through close collaborations with medical and scientific communities. Although Leica was initially dedicated to microscopy and optics, it entered the field of neurosurgery in the late 20th century. In 1997, Leica introduced the M500 N, its first surgical microscope designed explicitly for neurosurgery [40]. Since then, Leica has continually advanced its neurosurgical microscopes in digital visualization and ergonomic features, thereby becoming a pivotal player in modern neurosurgery.

Current Leica models: The currently available Leica surgical microscope models are the M525 F20 [5], the M530 OHX for spine [41] surgery (only in the United States and Canada), the M530 OHX for microsurgery [42] (only outside the United States and Canada), the PROvido [43,44], the ARveo 8 [45-47], and the Evolved ARveo 8 (Table 2) [5,42-46,48-57]. However, this availability may vary over time, so individuals should contact the local company representative for the most current information.

**Table 2:** Technical specifications of surgical microscopes and exoscopes from Leica, Mitaka, and Synaptive B: blue, Exo: exoscope, FL: fluorescence, FL-Y: fluorescence of indocyanine green in the yellow spectrum, ICG: indocyanine green, LED: light-emitting diode, Micro: microscope, Microsurg: microsurgery, NA: not applicable, NS: not specified, V: volts, VA: volt-ampere, Y: yellow, 3D: three-dimensional, 5-ALA: 5-aminolevulinic acid. ^a^Hybrid: a microscope equipped with digital 3D functionality, allowing it to operate both as a traditional optical microscope and as an exoscope.

Feature	Leica							Mitaka			Synaptive
	M525 F20 [5]	M530 OHX (Premium) [42]	M530 OHX (Precision) [49,57]	M530 OH6 [50]	ARveo 8 [45,46]	Evolved ARveo 8[48]	PROvido [43,44]	MM90 [52]	HawkSight [53,54]	MM77 [55]	Modus X [56]
												Type	Micro	Micro	Micro	Micro	Hybrid^a^	Hybrid^a^	Micro	Micro	Exo	Hybrid^a^	Exo
Power consumption	100–240 V	100–240 V	100–240 V	100–240 V	100–240 V	100–240 V	100–240 V	NS	NS	NS	120 V or 230–240 V or 220 V
	50–60 Hz	50–60 Hz	50–60 Hz	50–60 Hz	50–60 Hz	50–60 Hz	50–60 Hz				50–60 Hz
	500 VA	1600 VA	1600 VA	1600 VA	1300 VA	1300 VA	800 VA				
Weight, kg	229	320	320	320	320	320	370	250	210	250	320
Magnification	1.2× to 12.8× with 10× eyepiece	1.0× to 12.1× with 10× eyepiece	1.0× to 12.0× with 10× eyepiece	1.0× to 12.1× with 10× eyepiece	1.0× to 12.1× with 10× eyepiece	1.0× to 12.1× with 10× eyepiece	1.0× to 12.1× with 10× eyepiece	Optical zoom: 8:1	NS	Optical zoom: 10:1	Optical zoom: ≤7× Digital zoom: ≤40%
											Total zoom range: ~9.8×
Eyepieces	10×	10×	10×	10×	10×	10×	10×	10×	NA (exo only, no oculars)	10×	NA
	12.5×	8.3×	8.3×	8.3×	8.3×	8.3×	8.3×				(3D monitors and polarized eye shields)
	-	12.5×	12.5×	12.5×	12.5×	12.5×	12.5×				
Fluorescence dyes	None	FL400	FL800	FL400	FL400	FL400	FL560	ICG	ICG	ICG	Modus B Modus Y
		FL560	-	FL560	FL560	FL560	FL800	5-ALA	5-ALA	5-ALA FL-Y	
		FL800	-	FL800	FL800	FL800	-	-	-	-	
		GLOW800	-	-	GLOW800	GLOW400	-	-	-	-	
		-	-	-	-	GLOW800	-	-	-	-	
Illumination	Dual 180-W xenon arc lamp via fiber optics	High-output dual 400-W redundant xenon arc lamp via fiber optics	High-output dual 400-W redundant xenon arc lamp via fiber optics	High-output dual 400-W redundant xenon arc lamp via fiber optics	High-output dual 400-W redundant xenon arc lamp via fiber optics	Two 400-W xenon arc lamps with their own power supply	300-W xenon arc lamp with 75-W LED lamp backup or dual xenon arc lamps	400-W xenon arc lamp	LED	400-W xenon arc lamp	Adjustable white LED, variable preset brightness in optical mode
Working distance, mm	207–407	225–600	225–600	225–600	225–600	225–600	225–600	200–600	200–1000	200–650	300–650

The M525 F20 surgical microscope, equipped with the M525 optics carrier, is the most advanced model in the M500 series [40]. After the release of the neurosurgery-specific model (M500-N), the microscope underwent continual advances, culminating in the launch of the M520 optics carrier in October 2003 and eventually, the introduction of the M525 optics carrier in September 2006. Among the various stand systems available for Leica microscopes, the F20 floor stand is currently standard on the M525 model [5,58].

The M525 F20 is a compact, maneuverable microscope with enhanced ergonomics. It features a 6:1 motorized zoom and a multifocal lens. Wide-field eyepieces accommodate users who wear eyeglasses [58]. The illumination field adapts automatically to the zoom level but can also be adjusted manually. Safety features such as AutoIris and BrightCare automatically adjust the illumination field and intensity based on the zoom level and working distance, thereby minimizing the risk of tissue damage [58]. These integrated mechanisms enhance both surgical safety and user convenience [5].

The M525 F20 supports various accessories, including a dual stereo observer attachment and a rotatable beam splitter with adjustable viewing ratios [58]. It features ergonomic binocular options (30°-150° adjustable or fixed 45°), a C-mount video adapter with 3:1 zoom, and a 35-100-mm focal length. The system is compatible with sterilizable components and can be integrated with commercially available lasers and shutters.

The M525 F20 differs from earlier Leica models in its stand configuration, although all models share the same optics carrier [59-64]. The older models were tailored for neurosurgical use with advanced stabilization features, whereas the M525 F20 offers a more compact, versatile design for broader surgical applications [5,60]. The M525 optics carrier is compatible with various stand types, including the M525 F40, M525 F50, M525 OH4 (designed by Mitaka), and M525 CT20 (a ceiling-mounted stand, alternative to the M525 F20), to accommodate different surgical requirements [62-64].

**Figure 3:**
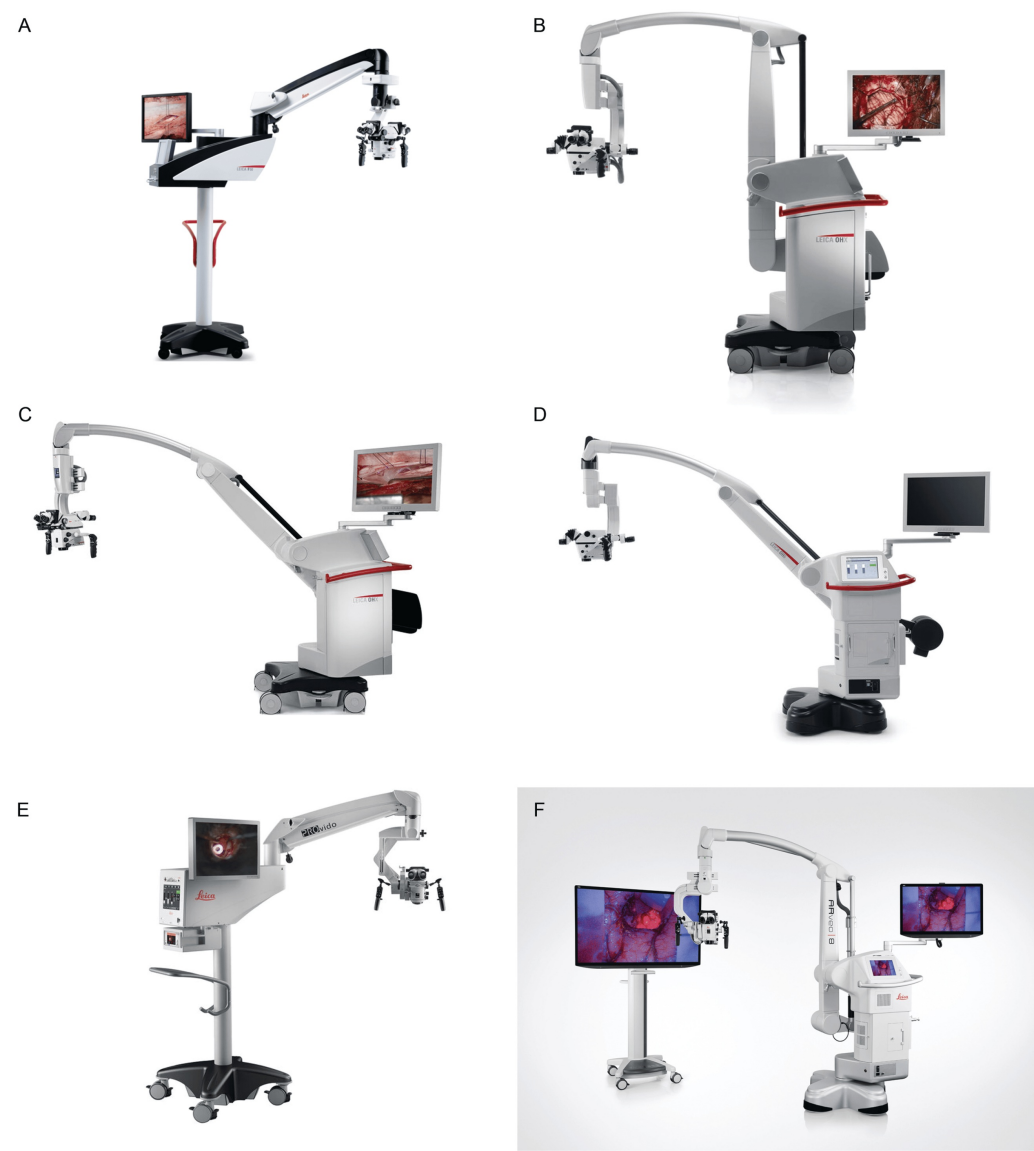
Currently available surgical microscopes from Leica Microsystems GmbH (Wetzlar, Germany (a subsidiary of Danaher Corp., Washington, DC)) that are actively used in neurosurgery (A) M525 F20 [5]. (B) M530 OHX Premium Surgical Microscope [42]. (C) M530 OHX Precision Surgical Microscope [57]. (D) M530 OH6 [50]. (E) Leica PROvido [44]. (F) Evolved ARveo 8 [48]. Used with permission from Leica Microsystems.

The Leica M530 series, including the M530 OHX premium surgical microscope [42], was introduced in 2014 to deliver advanced optical solutions for neurosurgery, including FusionOptics, apochromatic optics, and 400-W xenon illumination for improved visualization in deep surgical fields [41,49]. The M530 OHX premium [42] and the M530 OHX precision surgical microscopes remain available outside the United States and Canada, but the M530 OH6 [42,50] has been succeeded by the ARveo 8 [45,46] and Evolved ARveo 8 [48] models (Figure 3) [5,42,47,50,57,65].

The M530 series of microscopes is tailored for neurosurgery and spine procedures, combining optical precision with ergonomic design. Models such as the M530 OH6 and M530 OHX use FusionOptics, which combines high resolution with extended depth of field to enhance image clarity [42,50,66]. Additionally, the small-angle illumination (SAI) feature improves visibility in deep, narrow surgical fields by reducing shadows and enhancing depth perception.

The Leica M530 OHX and M530 OH6 microscopes use FusionOptics technology, delivering enhanced depth of field and resolution to both the primary and assistant surgeons via stereo attachments; in the M530 OHX Precision model, this feature is limited to the primary observer [50,57]. The apochromatic optical system in these microscopes ensures high contrast and natural color by minimizing chromatic aberration. The microscopes feature a 6:1 motorized zoom. Their field of view varies from 17.4 mm to 210 mm and is adjustable through motorized or manual focus mechanisms [42,50,57]. Wide-field eyepieces and 360°-rotating adapters accommodate both primary and assistant surgeons. For enhanced stability, they are equipped with six electromagnetic floor brakes. The integrated SpeedSpot laser enables rapid, accurate microscope positioning, enhancing the surgical workflow.

**Figure 4:**
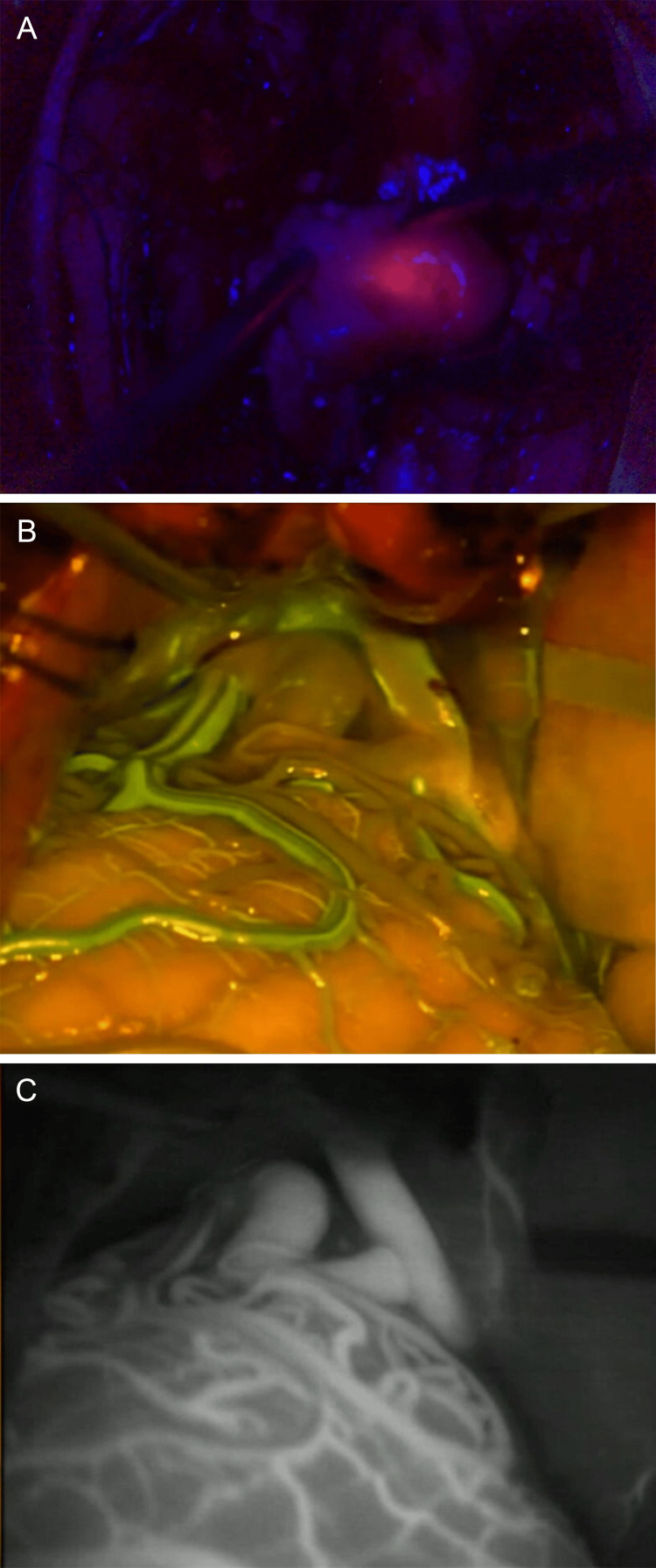
Intraoperative visualization with different microscopes from Leica Microsystems of pathologies with use of various fluorescent dyes and optical filters (A) Intraoperative glioma visualization using FL400 Blue Light Fluorescence Module (5-ALA) [67]. (B) Intraoperative visualization of an AVM using the FL560 fluorescein fluorescence module, compared with (C) visualization using ICG [68]. AVM: arteriovenous malformation, ICG: indocyanine green Used with permission from Leica Microsystems.

Compared with models such as the M525 F20, the M530 OHX and the M530 OH6 offer enhanced modularity through an OpenArchitecture design that supports integration with technologies such as IGS and laser systems [50,57]. Both the M530 OH6 and the M530 OHX premium surgical microscopes support multiple fluorescence modules (FL400, FL560, and FL800) (Figure 4) [67,68], whereas the M530 OHX precision surgical microscope is limited to FL800. Additional features include video recording and dual-imaging support for a second observer.

Launched in 2018 to replace the M720 OH5, the Leica PROvido remains in production as a multidisciplinary microsurgical microscope [44,51]. It integrates technologies from earlier models, including FusionOptics, SAI, AutoIris, and BrightCare Plus, that enhance both visibility and patient safety. The SpeedSpot laser further improves precision and workflow efficiency [65].

The PROvido microscope features a motorized zoom lens and a multifocal lens for precise focus and adjustable working distance, complemented by ergonomic enhancements, including wide-field eyepieces and a rotatable adapter [43]. A xenon arc lamp, optimized with SAI, provides illumination for improved depth visibility, and a backup lighting system ensures uninterrupted use. The PROvido microscope supports FL560 and FL800 fluorescence modules, but it does not include CaptiView image injection technology [43,65].

The PROvido microscope uses the same optics carrier as the M530 OHX series, with the main distinction being the design of the microscope stands [43,57,65]. The PROvido stand is larger and heavier, offering greater stability and vibration resistance, whereas the M530 OHX stand is more compact and maneuverable. Both systems feature six electromagnetic brakes, but the M530 OHX also includes automatic balancing and an automatic counterbalance/balance compensation mechanism for enhanced control.

The Leica ARveo 8 and Evolved ARveo 8, introduced in December 2021, are Leica’s most advanced surgical microscopes [45-48,69]. Like previous models, they incorporate technologies such as FusionOptics, SAI, SpeedSpot, AutoIris, and BrightCare Plus. The ARveo 8 series also introduces EnhancePath, which integrates IGS, endoscopy, and AR into the microscopic view, enabling surgeons to merge preoperative and intraoperative imaging to improve navigation and outcomes [46,47,70].

The ARveo 8 series also features the M530 optics carrier as on the M530 series of microscopes; thus, it has the same optical and illumination capabilities [41,46,47,49,69]. The primary distinction of the ARveo 8 series is the custom Mitaka-designed overhead ARveo stand and the integration of EnhancePath, which enables advanced IGS systems for improved surgical navigation [46,70].

Like the M530 OHX stand, the ARveo 8 stand features an antimicrobial-coated construction and six electromagnetic brakes, and it has similar height and reach specifications [46,47,70]. However, the ARveo 8 stand improves stability by using a larger base (720 × 720 mm vs 690 × 690 mm) and a heavier frame, and introduces an advanced motion system with six-axis balancing and vibration damping. It also includes specialized spine and cranial braking modes to optimize positioning for different surgical contexts.

EnhancePath is a key innovation in the ARveo 8 series that seamlessly integrates with Brainlab IGS to combine digital and surgical visualization without external hardware [46,47,70]. It updates preoperative imaging in real time, supports augmented reality (AR) fluorescence for enhanced anatomical and functional guidance, and displays IGS data via picture-in-picture navigation, allowing surgeons access to critical intraoperative information without diverting from the surgical field.

**Figure 5:**
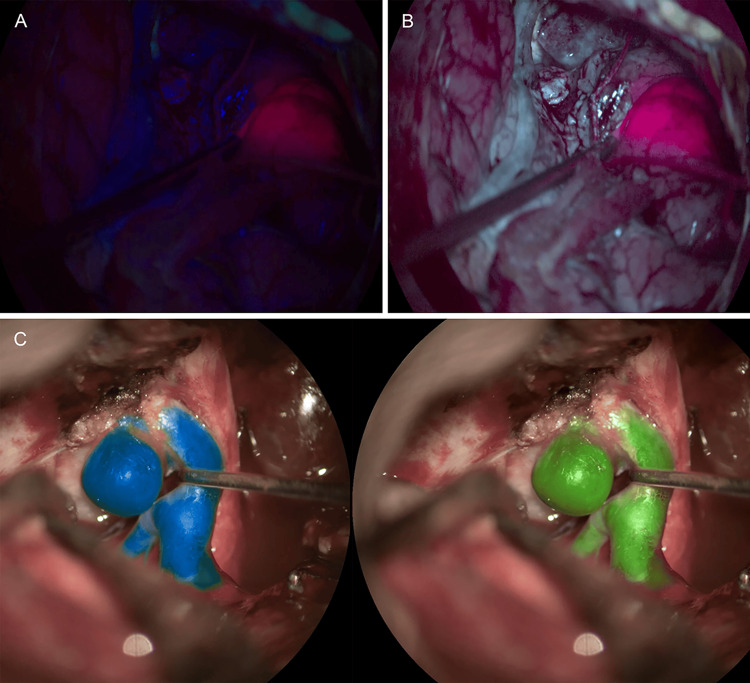
Intraoperative images captured using the FL400, GLOW 400, and GLOW800 AR image-injection modules integrated into surgical microscopes from Leica Microsystems (A) Intraoperative glioma visualization using FL400 (5-ALA) AR fluorescence [67]. (B) Intraoperative 3D glioma visualization with the GLOW400 AR module, generated from the FL400 (5-ALA) fluorescent signal [71]. (C) The ability of the GLOW800 module to be used with different contrasts and color variations aids intraoperative aneurysm clipping [72]. AR: augmented reality, 5-ALA: 5-aminolevulinic acid, 3D: three dimensional. Used with permission from Leica Microsystems.

The Evolved ARveo 8, launched in January 2024, builds on the ARveo 8 with enhanced AR visualization and expanded fluorescence imaging capabilities [48,70]. In addition to FL400, FL560, and GLOW800, it supports FL800 and GLOW400, enabling broader staining options and improved visualization of high-grade gliomas (Figure 5) [46,47,67,70-72]. The entire Leica AR platform now operates in 3D, which enhances depth perception and spatial awareness [47,70].

**Figure 6:**
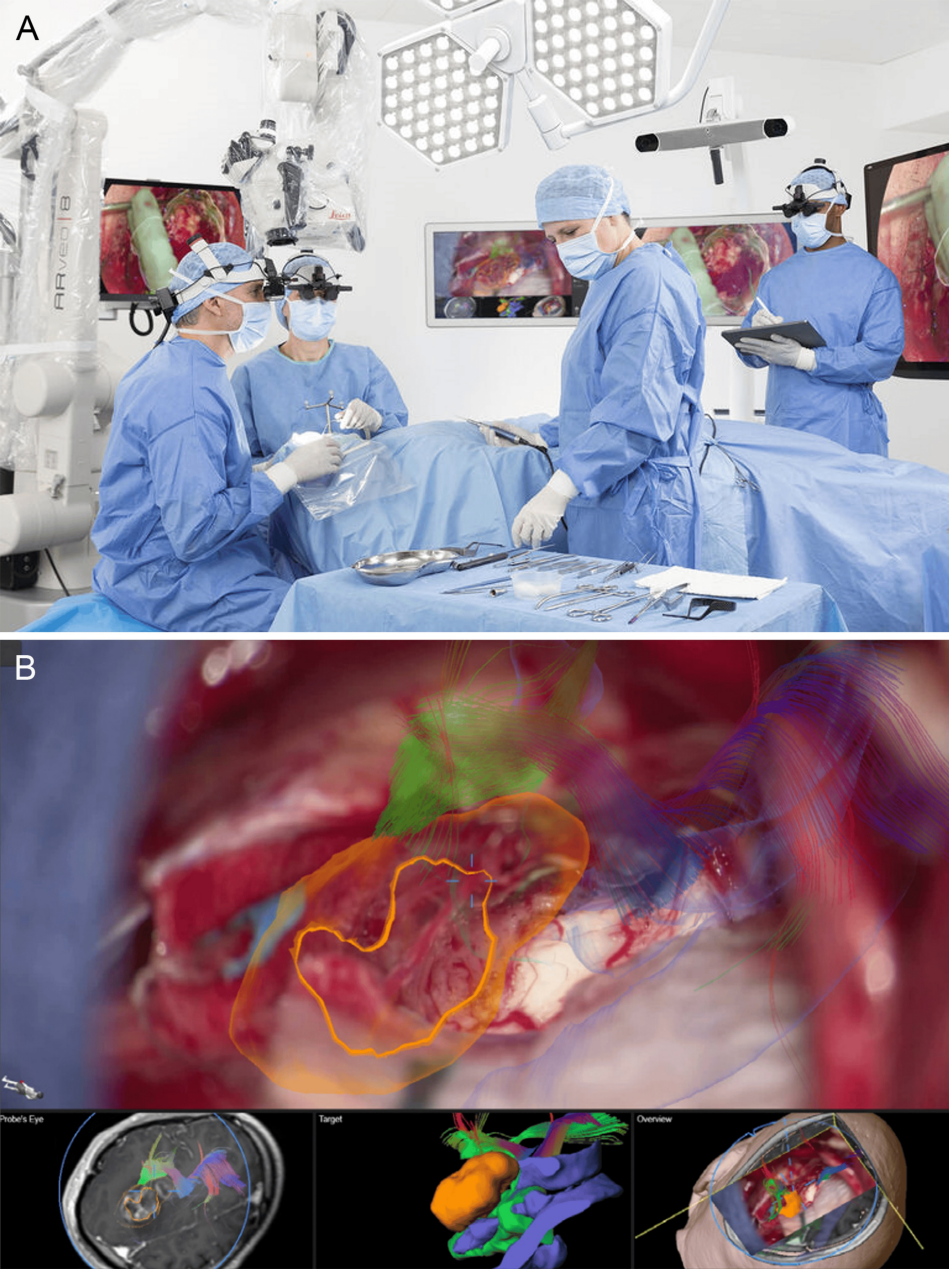
Educational role of products from Leica Microsystems GmbH is demonstrated both through laboratories equipped with training microscopes and through advanced intraoperative visualization tools that support the surgical team (A) The MyVeo surgical visualization headset enables the surgical team to visualize the operative field from the perspective of the surgeon [73]. (B) Segmentations and tractographies are integrated into intraoperative images via the image-guided surgery system of the Evolved ARveo microscope [47]. Used with permission from Leica Microsystems.

Features of Leica microscopes: Leica surgical microscopes combine high-end optical clarity with ergonomic design. They offer advanced features like FusionOptics, SAI, and seamless integration with AR and IGS systems, ensuring precise visualization while maintaining intuitive surgeon control. Like other companies, Leica also places particular emphasis on surgical training. This commitment is highlighted by the ability of surgical assistants to use the MyVeo headset (Figure 6A) [73], which was developed for the ARveo 8 series, to follow the procedure from the perspective of the surgeon and the introduction of the M320 T microscope, which was designed explicitly for surgical training. Although evolving IGS systems provide considerable benefit to surgeons, they also enhance the ability of novice surgeons to better understand the anatomy during operations (Figure 6B) [47]. Tools such as the MyVeo headset enable them to experience AR systems in real time from a first-person, 3D perspective, thereby further enriching their surgical expertise.

Leica has gained considerable attention through an innovation in its fluorescence modules. The GLOW800 AR fluorescence mode, integrated into the ARveo 8 model, enables simultaneous visualization of cerebral anatomy and blood flow under white light. In other words, this mode eliminates the need to switch back and forth to reconcile black-and-white near-infrared images. This advancement is particularly beneficial in surgeries involving arteriovenous malformations, aneurysms, bypasses, and microvascular decompression [69].

Leica also offers a wireless foot switch, an accessory for its surgical microscopes that allows hands-free control of microscope settings and enables direct intraoperative adjustments by foot [74]. Users can preset imaging parameters in the control panel and modify them during surgery with minimal disruption. The wireless design allows flexible placement in the operating room, reducing cable clutter and enhancing workflow efficiency.

Mitaka (Mitaka Kohki Co., Ltd.)

**Figure 7:**
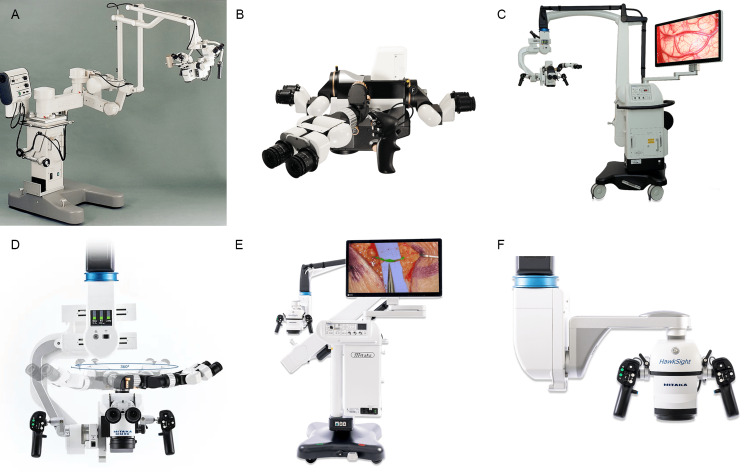
Currently available microscope models by Mitaka Kohki Co., Ltd. (Tokyo, Japan) that are actively used in neurosurgery (A) The Space Pointer Cygnus Microscope was developed in 1988 in collaboration with Leica Microsystems GmbH (Wetzlar, Germany (a subsidiary of Danaher Corp., Washington, DC)) [76]. (B and C) The MM77 surgical microscope provides ergonomic options for three simultaneous observers [55]. (D) The high-resolution MM90 surgical microscope can achieve an 8:1 zoom [52]. (E and F) HawkSight surgical microscope [53]. Used with permission from Mitaka USA.

Mitaka, founded in 1966 by Giichi Nakamura, began as a developer of high-precision telescopes and optical measurement instruments for academic institutions [75]. In 1988, the Nakamura brothers at Mitaka developed their first neurosurgical microscope, the stereotactic Space Pointer Cygnus, in collaboration with Leica to meet the needs of neurosurgeons [76,77]. A notable innovation was an overhead positioning stand that placed the microscope above the surgeon and the body of the device behind the surgeon, thereby improving intraoperative mobility [78]. Mitaka subsequently built a strong product line, continually refining the Cygnus model and developing advanced models (Figure 7, Table 1) [52,53,55,76,79].

Current models: The Mitaka MM77 is designed to enhance precision and ergonomics (Table 2) [55]. It features a 10:1 zoom ratio, a 30 mm-diameter zoom aperture for both left- and right-hand zoom channels, and an optical resolution of 135 lp/mm, providing surgeons with clear, detailed images. The working distance ranges from 200 mm to 650 mm, with a 98-mm field of view at 200 mm and an on-screen resolution for high-quality, real-time feedback at 90 lp/mm [80]. The compact design accommodates up to three simultaneous observers, promoting collaborative procedures [55].

The Mitaka MM90 model emphasizes ergonomic design tailored for seated operations (Table 2) [52]. With its short ocular-corpus length of 270 mm, the MM90 reduces fatigue during extended procedures [52,81]. It features an 8:1 zoom, 22× magnification, 400-W twin xenon lights, an integrated 4K camera, and a deep-diode indocyanine green (ICG) overlay for high-resolution recording and fluorescence imaging.

The MM90 also features multiple analog control methods, including a control panel (the surgeon’s liquid-crystal display), a foot pedal, a mouth switch, hand-grip controls, and a touchscreen for video recording [52]. Moreover, the microscope is mounted on a Mitaka overhead stand that enables automatic balancing with a single button [82].

The Mitaka HawkSight is the only 8-sensor 4K 3D video surgical exoscope available, with a dual 4-sensor camera for native 4K 3D resolution (Table 2) [53,79]. With a 200-1000-mm working distance and quick autofocus, it provides advanced imaging and a broad yet detailed surgical view. It also supports three fluorescence modes, enabling simultaneous observation of visible and near-infrared light, which is best for ICG fluorescence imaging [53]. However, the complexity and advanced features of HawkSight may require additional training, as evidenced by the establishment of the Mitaka Europe Training Center, which includes HawkSight in its inventory [83].

Features of Mitaka microscopes: Mitaka excels in fluorescence imaging with a two-stage system for fluorescein and 5-ALA, which allows the surgeon to toggle between high sensitivity and contrast [84]. The Mitaka HawkSight exoscope integrates a unique camera with four sensor chips and deep-diode near-infrared technology that improves sensitivity, signal-to-noise ratio, and overlay accuracy, which is a technology not currently used by other manufacturers [54]. Mitaka is also known for its reliability, compartmentalized functionality, and internal operating systems that support patient-critical features [82].

Mitaka’s surgical microscopes are distinguished by their ease of movement and positioning, which is critical, particularly for neurosurgeons who rely on mouth switches for precise control. Mitaka stands out in mobility and ergonomics. Its zero-weight counterbalance stand, used in models such as the HawkSight [54] and MM51 [85], allows smooth, precise repositioning without the need for focal length extenders. Ergonomically, Mitaka is the only manufacturer to offer horizontal optics in its MM77 and MM90 models, minimizing the ocular-corpus distance and enabling a comfortable position across various procedures and surgeon heights. In 2020, Goehre et al. [81] reported that the MM90 model has the shortest ocular-corpus length at 270 mm, which is about 12% shorter than that of other microscopes, making it preferable for seated microsurgical procedures.

Mitaka also manufactures microscope stands for microscopes from well-known companies, such as the Leica ARveo 8, with a focus on simplicity and ease of use. For example, HawkSight enables a focal depth adjustment with a single button press. This versatility allows surgeons to select the optimal configuration based on the patient's surgical needs, thereby improving efficiency. Moreover, the choice of analog controls is purposeful. Mitaka uses Controller Area Network (CAN) bus technology, a highly reliable data communication protocol for touchscreen controls.

Finally, Mitaka is known for its ease of use with analog, user-friendly interfaces, offering intuitive operation with minimal training. For example, the MM90 model offers high-speed zoom and simplified controls, eliminating touchscreens and complex software, thereby enabling medical personnel to master the system quickly without extensive instruction [52].

Olympus (Olympus Corp.)

Olympus, founded in Japan by Takeshi Yamashita, aimed to elevate Japanese microscopes to European standards. From its founding in 1919 through the late 1920s, Olympus developed a comprehensive range of microscopes, establishing a strong foundation for subsequent advances [86,87]. In the 1930s, Olympus introduced improvements, including binocular configurations and mechanical stages [30]. Olympus also diversified into camera optics with its first camera, the Semi-Olympus, in 1932, and it was renamed Takachicho Optical Industries in 1942 to reflect its optical focus [86]. Despite wartime challenges, Olympus continued to innovate and, after World War II, established a global presence.

**Figure 8:**
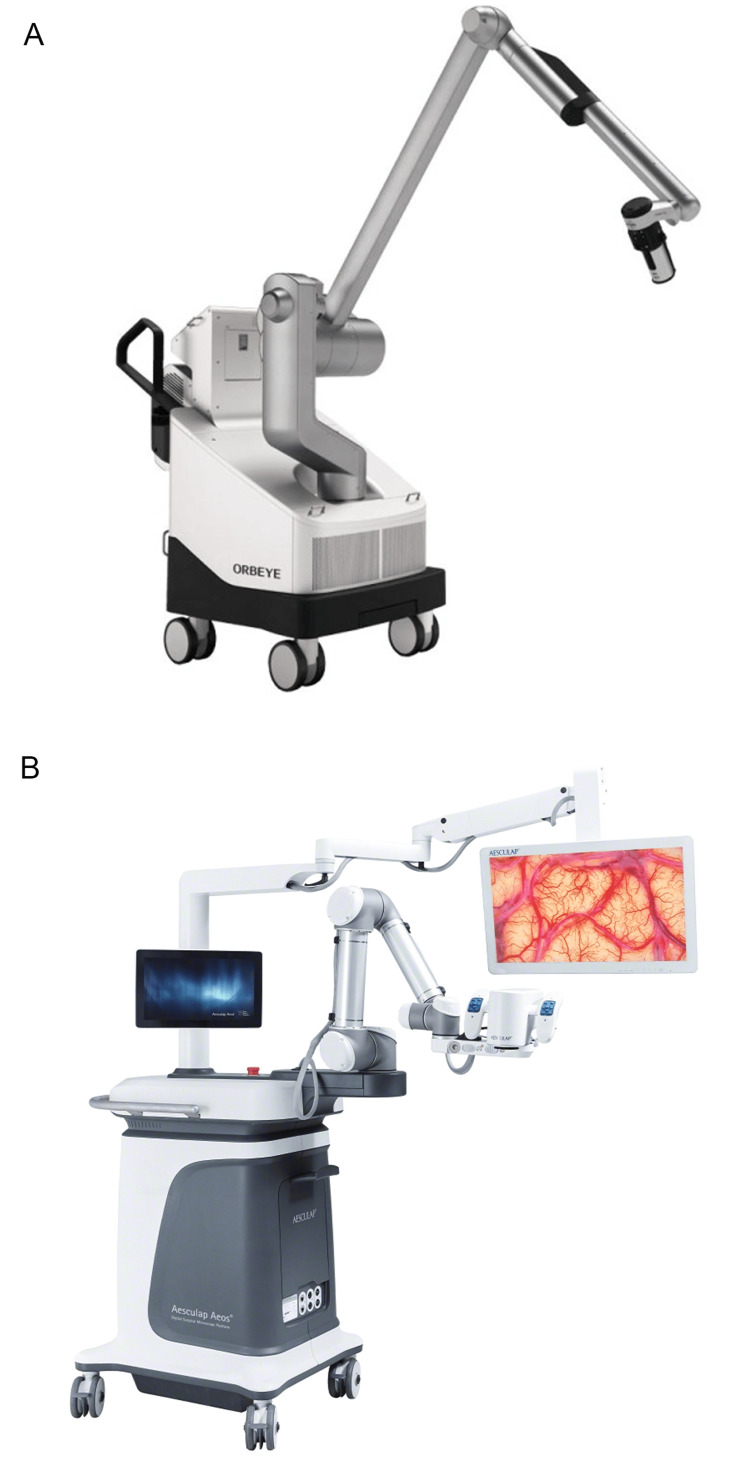
Exoscope models from Olympus and Aesculap that are actively used in neurosurgery (A) The Olympus ORBEYE exoscope is a 4K 3D orbital camera system [29]. (B) The Aesculap Aeos robotic exoscope [31]. Used with permission from Olympus and Aesculap.

Today, Olympus maintains a strong international presence, with subsidiaries and regional hubs in Europe, the Middle East, Africa, the Americas, and Asia [88,89]. In neurosurgery, Olympus offers a single model explicitly designed for neurosurgical procedures: the ORBEYE exoscope (Figure 8, Table 1) [29-31]. This flagship device was among the first developed by Sony Olympus Medical Solutions, Inc., a joint venture established in 2013 with Sony Corp. (Tokyo, Japan) to advance 4K 3D imaging technologies for surgical applications by combining the expertise of engineers from both companies. The ORBEYE features a compact optical unit that enhances surgical ergonomics and provides high-resolution 4K 3D visualization with excellent depth perception [29]. This exoscope offers several notable advantages in surgical settings. Its compact, flexible optical unit allows various surgical positions without compromising the surgeon's posture, enhancing ergonomics compared to traditional microscopes with ocular lenses [90,91]. Its large 4K screen facilitates teamwork and surgical training, whereas its wireless foot switch streamlines control [29]. In addition to its optical benefits, the ORBEYE system integrates advanced imaging modes, including near-infrared and blue light, which support vascular and tumor visualization, respectively, during procedures [92]. The near-infrared mode delivers high-brightness ICG fluorescence without filter dependence, whereas the blue-light mode enhances fluorophore-based tissue contrast under blue excitation. The ORBEYE features optical and digital zoom up to 26×, agile autofocus, and zero image latency, ensuring seamless synchronization between hand movements and screen display. These features collectively support improved safety and precision during microsurgical manipulation.

However, the rotated view of the ORBEYE can hinder surgical assistants. Furthermore, its steeper learning curve makes its use less intuitive than that of conventional microscopes [91]. Nonetheless, the ORBEYE could ultimately replace traditional microscopes due to its immersive experience and ergonomic advantages during complex, prolonged neurosurgical procedures.

Aesculap (Aesculap, Inc.)

Aesculap was founded by Gottfried Jetter in 1867 in Tuttlingen, Germany [93]. Today, it is one of the world’s largest surgical instrument manufacturers, with its instruments widely used in neurosurgery. Aesculap’s production of microscopes, endoscopes, and exoscopes is relatively recent, given its long-standing history of surgical instrument manufacturing. The most widely used Aesculap model in neurosurgery today is the Aeos Robotic Digital Microscope (Table 1) [31].

The Aeos offers heads-up positioning, hands-free foot switch control, 10× optical zoom, 3D 4K imaging, and robotic assistance with six degrees of freedom [31]. The Aeos model stands out by combining the advantages of robotic-assisted movements with the ergonomic benefits of heads-up operation. Its robotic features, such as lock-on target (enabling pivoting around a fixed point of interest) and waypoints (saving and returning to previously defined positions), further enhance surgical precision and workflow efficiency [31]. Its AR capabilities in ultraviolet light mode provide additional insights from combined fluorescence and white light images. The Aeos also integrates seamlessly with neuroendoscopes and other imaging technologies.

The use of the Aeos for microsurgical applications has been deemed both feasible and safe [3]. In 2024, Calvanese et al. [94] reported that the Aeos was safe and that neurosurgeons and assistants alike benefited from shared 3D intraoperative views. In a 2022 pilot study, Gabrovsky et al. [95] identified similar benefits for experienced neurosurgeons who transitioned from a conventional operative microscope to the Aeos robotic digital microscope (e.g., it reduced physical strain and frustration). Motov et al. [96] also demonstrated that the use of the Aeos in minimally invasive spinal surgery yielded benefits such as enhanced visualization, precision, and maneuverability. The integration of the Aeos microscope into surgical education also provides an effective learning platform for residents and junior surgeons [97].

Synaptive (Synaptive Medical, Inc.)

Synaptive Medical was founded by Wes Hodges and Cameron Piron in 2012 in Toronto, Canada [98]. In 2015, the US Food and Drug Administration approved Synaptive’s first surgical visualization system, the BrightMatter Guide, a neuronavigation system that displays 3D tractographic images of the brain. BrightMatter technology was first installed in 2016 at Mount Sinai Hospital in New York City [99]. The following year, Synaptive released Modus V, a robotic digital exoscope [100]. Modus V incorporates advanced high-powered 4K 3D optics, 27× magnification, voice-activated control, autofocus, and integration with surgical planning via the BrightMatter platform [100-102].

Synaptive’s latest model is the Modus X robotic exoscope, which was launched in 2023. Features of the Modus X include 4K 3D optics, 27× magnification, automated image focus, voice guidance, and FGS capabilities [56]. Fluorescence options include blue light for real-time tissue differentiation and near-infrared fluorescence with ICG for better visualization of blood vessels and circulation.

Advantages of exoscopes

First introduced in neurosurgery in the early 2000s, exoscopes offer several advantages over traditional microscopes, including improved ergonomics, enhanced visualization, a heads-up display for education, greater camera flexibility, and reduced operative time [103-109]. Moreover, the use of exoscopes in spine surgery has been associated with reduced blood loss, shorter procedures, and shorter hospital stays [108,110]. The Olympus ORBEYE and the Zeiss Kinevo 900 excoscopes are preferred in neurosurgery for their superior image quality and ergonomic benefits [106,111].

Recent exoscope models provide 4K 3D high-definition visualization, improving depth perception and magnification during intricate procedures [103,107]. Their ergonomic design supports a natural posture and reduces neck strain. Shared viewing platforms enhance training and intraoperative collaboration [105,106]. Exoscopes are versatile and have been used in tumor resections and in vascular and spinal procedures; they can also be integrated with AR and robotics [112,113]. The learning curve for using an exoscope is typically short, with a smooth transition from traditional microscopes [103,106,114]. However, limitations include reduced depth perception, potential image-quality issues in deep fields, manual focusing, higher cost, and visual artifacts, and these limitations could be especially pronounced for older surgeons or in complex anatomical areas [108,109].

Digital 3D exoscopes also offer high-definition visualization without the need for eyepieces. A comparative study found that although exoscopes and standard microscopes provide similar procedural quality, exoscopes offer higher magnification and improved ergonomics but require more time to use [115]. These advances underscore the evolving role of hybrid microscopes and exoscopes in neurosurgery, where they offer improved visualization, precision, and ergonomic benefits, thus potentially contributing to better surgical outcomes [116].

Discussion

Since the first use of operative microscopes in neurosurgery, advances in magnification, digital visualization, fluorescence imaging, and robotic assistance have positioned them as essential tools in modern neurosurgery. Leading manufacturers offer unique features tailored to specific surgical applications.

Advantages and Limitations of Currently Available Neurosurgical Operative Microscopes

The advantages and limitations of the six leading microscope brands can be summarized by highlighting their most innovative features.

Zeiss microscopes, particularly the Kinevo 900 S and the Pentero 800 S, are known for their high optical quality, AI-assisted positioning, digital hybrid visualization, and broad fluorescence options (Blue 400, Yellow 560, Infrared 800) [21,22,36]. Zeiss is a leader in AI automation, fluorescence integration, and digital-robotic fusion, making its microscopes among the most versatile systems for neurovascular and tumor surgery. Potential downsides include cost and the need for specialized training.

Leica microscopes offer apochromatic optics, FusionOptics, and a compact design with OpenArchitecture for integration with IGS and AR [47,50]. Leica is also known for its wide range of fluorescence options (FL400, FL560, and FL800) and its image injection modules (GLOW400 and GLOW800). The ease of use and high optical clarity of Leica microscopes make them among the most widely used in microneurosurgery. As Leica’s emphasis on optical clarity and ease of use suggests, the company’s primary focus is on optimizing optical performance and seamless integration with surgical navigation systems rather than on incorporating AI-based positioning into its latest models.

Mitaka specializes in fluorescein and 5-ALA imaging, dual-stage contrast systems, and the data communication protocol controller area network bus reliability. The MM90 model supports excellent maneuverability and surgeon ergonomics [52,82,84]. It is also preferred for fluorescence-guided tumor visualization. Additionally, Mitaka’s neurosurgical microscopes are relatively small compared to others, which can be advantageous in crowded operating room settings. However, they lack AI automation and digital visualization, which may limit their use in advanced workflows.

Olympus has introduced the ORBEYE exoscope, a 4K 3D digital exoscopy system that eliminates the need for eyepieces, improving ergonomics and enabling shared team views [29]. However, this microscope has a steep learning curve and presents monitor orientation issues for surgical assistants.

Aesculap integrates robotics, AI automation, and AR in its Aeos robotic microscope, thereby enabling digital manipulation during surgery [31]. The Aeos's limitations include its high cost and extensive training requirements.

Synaptive Medical offers several advanced visualization capabilities for neurosurgery in its Modus X exoscope, including 4K 3D optics, fluorescence visualization with blue-light and near-infrared modalities, and a patented 4K ICG Image Fusion for real-time contrast of microvessels against background anatomy [56,117]. Other features include the Modus X's ergonomic design and its integration with operating room systems. The Synaptive exoscope integrates navigation, robotic automation, and data analytics for improved surgical workflow [98]. However, as with other exoscopes, its limitations include high cost and the need for training.

For these six leading microscope brands, ergonomics and optical positioning vary. Zeiss and Mitaka microscopes optimize overhead usability, whereas Olympus microscopes improve surgeon posture by eliminating the eyepiece (at the expense of reduced visibility for surgical assistants) [83]. Zeiss microscopes continue to lead in multifunctional surgical environments, whereas Leica and Mitaka microscopes serve focused niches. The Aesculap and Synaptive systems show promise for the future of robotic and AI-assisted microsurgery [31]. The Olympus, Aesculap, and Synaptive systems all emphasize exoscopic and robotic designs that support ergonomic workflows.

In a 2022 survey, 75% (71 of 95) of neurosurgeons reported using Zeiss microscopes, reflecting the company’s historical influence and technological longevity [118]. Zeiss models, such as the KINEVO 900 S and the PENTERO 800 S, dominate the market owing to their AI-driven automation, fluorescence versatility, and robotic features. Leica models remain strong for general use, while Mitaka models excel in fluorescence-based imaging and ergonomics. The Aesculap robotic exoscope shows promise but may be constrained by adoption barriers. As the field advances, innovations in AI, AR, and digital visualization are expected to continue refining precision, workflow, and clinical outcomes.

Future Directions and Limitations of the Study

The recent emergence of technologies such as AR and AI has launched a new era of innovation [119-121]. As manufacturers have incorporated these technologies into their microscopes, intraoperative performance and surgical training have improved. In parallel, the substantial increase in research interest in AR- and AI-driven microsurgical tools has further accelerated developments [119,122,123].

The integration of AR-based simulation platforms directly into surgical microscopes holds considerable potential. These systems enable residents and early-career surgeons to engage in interactive, anatomy-focused training within a realistic, immersive environment, offering them an operative experience without patient risk. This type of real-time, high-fidelity simulation may help bridge the gap between theory and practice in neurosurgery education. Moreover, AI has the potential to revolutionize ergonomic functionality. For example, AI-guided systems that track the surgeon’s head movement and gaze could enable dynamic microscope positioning, predictive focusing, and automated angle adjustments. Such intelligent control systems would reduce the need for manual repositioning, minimize workflow interruptions, and potentially shorten operative time, thereby improving efficiency and comfort during surgery.

Future research must address several challenges, including standardizing and clinically validating AR and AI tools, developing cost-effective implementations suitable for low-resource settings, and improving usability across various surgical workflows. Although high-end operating microscopes are essential tools for modern microneurosurgery, their global availability remains unevenly distributed. In many LMICs, the acquisition of these systems is constrained by financial constraints, and even when microscopes are available, access to maintenance and repair services may be insufficient, leading to reliance on outdated or non-serviceable models, often concentrated only in a few tertiary centers [32]. Improving global accessibility, long-term support, and sustainability of advanced surgical visualization systems is therefore an important goal. Initiatives such as the Madison Microneurosurgery Initiative, which helps provide microscopes to low-resource centers, highlight potential pathways to expand equitable access [124].

Future studies should also evaluate the effectiveness of integrated training modules within microscopes and their ability to enhance anatomical understanding, skill acquisition, and readiness for live surgery. Moreover, comparative studies assessing outcomes with and without AR- or AI-assisted systems are essential to determine their impact on surgical performance, patient safety, and learning curves.

Collaborative development involving engineers, clinicians, and educators is critical to ensure that future technologies are innovative, practical, accessible, and impactful across global health care systems. User-centered design must also be prioritized. Surgeons' feedback on usability, fatigue, cognitive load, and workflow integration should guide future product development. As these research needs are addressed, the next generation of surgical microscopes will no doubt become more intelligent, accessible, and impactful across diverse clinical environments.

Although this review aims to compile and synthesize all currently available manufacturer-based information and published literature on neurosurgical operative microscopes, several limitations should be acknowledged. First, much of the technical data is derived from publicly available manufacturer specifications and materials. Although these sources are authoritative, they may not accurately reflect device performance in real-world surgical conditions. Second, there is a lack of direct head-to-head comparative studies or randomized evaluations of microscope models, which limits the ability to draw evidence-based conclusions about superiority or clinical outcomes. Finally, although every effort was made to present the information objectively, the reliance on manufacturer-disclosed specifications introduces the possibility of inherent commercial bias. These limitations highlight the need for future independent comparative trials and multicenter user-based performance evaluations to guide surgical decision-making and procurement.

## Conclusions

The evolution of neurosurgical operative microscopes has led to remarkable advances in imaging quality, ergonomic design, and AR integration, all of which are pivotal to enhancing surgical precision and patient safety. Although state-of-the-art models from major manufacturers continue to push technological boundaries, many older or refurbished systems remain in use worldwide, particularly in resource-limited settings. This diversity reflects not only regional economic disparities but also varied clinical needs. Integrating advanced features such as AR visualization, wireless control interfaces, and dynamic viewing systems represents a promising direction for future innovations. However, more comparative studies and long-term evaluations are needed to optimize these technologies and ensure their broad applicability across different neurosurgical environments. Ultimately, our comparison of the currently available neurosurgical microscopes underscores the importance of continuous technological refinement to support both surgical excellence and comprehensive neurosurgical education.
